# Does Radiofrequency Echographic Multi-Spectrometry (REMS) perform similarly to Dual-energy X-ray Absorptiometry (DXA) in terms of Trabecular Bone Score (TBS) and FRAX?

**DOI:** 10.20945/2359-4292-2023-0456

**Published:** 2025-02-08

**Authors:** Débora Meira Ramos Amorim, Eliane Naomi Sakane, Sergio Setsuo Maeda, Marise Lazaretti-Castro

**Affiliations:** 1 Departamento de Medicina, Divisão de Endocrinologia, Universidade Federal de São Paulo, São Paulo, SP Brasil

**Keywords:** Radiofrequency echographic multi-spectrometry (REMS), dual-energy X-ray absorptiometry (DXA), trabecular bone score (TBS), FRAX

## Abstract

**Objective:**

To evaluate whether bone assessment by radiofrequency echographic multi-spectrometry (REMS) is influenced by trabecular bone integrity by comparing it to dual-energy X-ray absorptiometry (DXA) and the trabecular bone score (TBS). Additionally, the study aims to determine if comparing fracture risk using FRAX and the National Osteoporosis Guideline Group (NOGG) using the T-score from each method would lead to differences in a Brazilian female population.

**Subjects and methods:**

A sample of women aged 30-80 underwent REMS and DXA scans of axial sites at the Hospital São Paulo, Brazil. Subsequently, TBS was applied to DXA exams. Clinical data were obtained from hospital records and phone interviews to calculate fracture risk.

**Results:**

Among the 343 participants enrolled, 213 had comparable lumbar spine exams by REMS, DXA, and TBS, and 166 had comparable hip exams by REMS and DXA. The correlation between lumbar spine bone mineral density (BMD) by REMS and the TBS was low (r = 0.27, *p* < 0.001), as was the correlation between DXA and TBS (r = 0.39, *p* < 0.001). No statistically significant difference was found between the TBS classifications of osteoporotic lumbar spine by REMS and DXA (*p* = 0.178). Fracture risk data by FRAX were obtained from 119 participants, with 92% receiving concordant NOGG classifications for major osteoporotic fracture risk from REMS and DXA (κ = 0.71 CI95% (0.54 to 0.89), *p* < 0.001), and 87% for hip fracture risk (κ = 0.58 CI95% (0.38 to 0.77), *p* < 0.001).

**Conclusion:**

REMS performed similarly to DXA in assessing trabecular integrity using TBS. Additionally, no statistically significant difference was observed in fracture risk assessment by FRAX based on NOGG recommendations.

## INTRODUCTION

Osteoporosis is the most prevalent bone disease, defined by bone mass reduction and microarchitecture impairment that compromise bone strength, leading to increased fracture risk. Its diagnosis has been based on the bone mineral density (BMD) measured by dual-energy X-ray absorptiometry (DXA) since the World Health Organization (WHO) defined osteoporosis diagnosis as a BMD 2.5 standard deviation or more below the average value of healthy young women (T-score) (^1^).

Although DXA has excellent accuracy and precision (^2^), it has several disadvantages, including high cost, lack of portability, and use of ionizing radiation (^3^). Additionally, BMD is affected by variations in bone size and can be falsely increased by degenerative changes, representing a suboptimal fracture predictor (^3,4^). Therefore, new methods have been developed as an alternative or a complement to DXA, seeking to increase accessibility and improve fracture prediction.

Radiofrequency echographic multi-spectrometry (REMS) is a portable technology based on the frequency-domain analysis of ultrasound backscattered signals from axial scans. The software analysis is focused on the spectra portion related to the trabecular layer, thereby preventing the potential interference of degenerative artifacts typically observed in the cortical bone layer (^5-7^). According to the percentage of analyzed spectra classified as “osteoporotic” and “healthy” after comparison to the database, the Osteoporosis Score is calculated and converted into BMD values through linear equations, with T and Z-scores derived through quantitative comparisons to the National Health and Nutrition Examination Survey curve. The REMS demonstrated high correlation and agreement with DXA, validating its use in diagnosing osteoporosis and predicting fractures (^8,9^).

Furthermore, the potential influence that REMS may be influenced by bone quality properties that are not included in the BMD measured by DXA has been discussed (8). This is based on method principles (^5,6^), a moderate correlation between the bone apparent integrated backscatter (AIB) measured by REMS and the trabecular bone volume fraction (BV/TV) by micro-computed tomography (^10^), and REMS ability to assess fracture risk (^9^), which has been shown to perform slightly better than DXA in discriminating participants with and without a previous fragility fracture in a multicentric study (^11^). Moreover, studies addressing comorbidities such as type 2 diabetes and anorexia nervosa (^12,13^) have explored the hypothesis that REMS may be useful in the assessment of impaired bone quality.

The trabecular bone score (TBS) is a tool employed in clinical practice that estimates bone microarchitecture integrity through a gray-level textural analysis applied to lumbar spine DXA images. Similarly to REMS, it is not influenced by lumbar osteoarthritis (^14^). However, the International Society for Clinical Densitometry (ISCD) stipulates that TBS can be used in association with BMD, though not as a standalone measure, to refine the fracture risk assessment (^15^).

The present study sought to evaluate the performance of REMS compared to DXA by examining their correlations with TBS and its accuracy in defining fracture risk probability as calculated by FRAX in a sample of adult women.

## SUBJECTS AND METHODS

### Study population

This study was conducted following the Declaration of Helsinki and was approved by the Scientific Committee and Research Ethical Commission of the Universidade Federal de São Paulo (Brazil) under the number 09713119.9.0000.5505.

The study population was recruited from June to August 2019 at the DXA Unit of Hospital São Paulo at the Universidade Federal de São Paulo for the first comparison between DXA and REMS for osteoporosis diagnosis in a Brazilian women population (^16^). The study employed a cross-sectional design, enrolling 343 women aged between 30 and 80 years with body mass index (BMI) < 40 kg/m^2^ referred for a DXA exam performed at Hospital São Paulo. Pregnant women, those unable to adopt an appropriate position, and those who declined to participate were excluded from the study. All participants underwent anthropometric assessments (weight, height, and BMI) and lumbar spine and hip scans by DXA and REMS during a single visit. Reports of each site were processed separately.

Poor-quality REMS and DXA exams were excluded from the study. In addition, participants who had BMI > 37 kg/m^2^ were excluded to prevent any potential interference with TBS accuracy (^17^), and those aged < 40 years due to FRAX risk calculation (^18^). Electronic medical reports were reviewed for clinical characterization of the population, with particular attention to ethnicity, menopausal status, comorbidities, and use of medications. A post-evaluation phone call was conducted to obtain information regarding the participants’ personal history of fragility fractures and the history of hip fractures among their parents. The FRAX risk was calculated using the tool available on the website page of FRAX-Brazil. The intervention thresholds were based on the NOGG (^19^) recommended by the Brazilian FRAX (^20^).

### Study design

The correlations of the lumbar spine BMD obtained by REMS and by DXA with the TBS index in a Brazilian adult women population were calculated, as well the distribution of TBS categories using both methods. The secondary objective was to compare the application of femoral neck T-scores obtained by REMS and by DXA in estimating the intervention thresholds by FRAX and adjusted by the National Osteoporosis Guideline Group (NOGG) strategy.

### Dual-energy X-ray absorptiometry

The BMD was measured at the lumbar spine (L1-L4), femoral neck, and total hip by a Discovery Wi device (QDR 4500, Hologic, USA). The reported least significant change (LSC) for the lumbar spine and total hip is 3.5% and 3.8%, respectively, in the DXA Service of Hospital São Paulo (^21^). Positioning and image acquisition were conducted following the ISCD protocol (^22^). BMD values (g/cm^2^) and their respective T-scores were obtained for diagnostic classification according to WHO criteria (^23^).

### Radiofrequency echographic multi-spectrometry

Two independent operators who had undergone training in this method performed all REMS acquisitions. They had at least four months’ prior clinical experience with REMS. The device used was an EchoStation model (Echolight Spa, Lecce, Italy) equipped with an echographic convex probe operating at the nominal frequency of 3.5 MHz, which detects unprocessed radio-frequency signals. The data processing methodology employed by the REMS technology has been described in previous papers, as well as the description of the patient positioning and image acquisition by the method (^5,6^). We only made one attempt to capture data at each site. The reported inter-operator REMS LSC is 3.96% for the lumbar spine and 5.35% for the femoral neck in the DXA Service of the São Paulo School-Hospital (^16^).

### Trabecular bone score

The lumbar spine TBS parameters were extracted retrospectively from DXA images using TBS iNsight Software (v. 2.2.0, Medimaps Group SA, Switzerland). In this study, we defined a TBS score ≥ 1.310 as indicative of normal microarchitecture, TBS between 1.230 and 1.310 corresponds to a partial degradation, and TBS ≤ 1.230 as degradation (^24^).

### Statistical analysis

We employed the Kolmogorov-Smirnov test to evaluate normality. Data exhibiting normal distribution were expressed as mean (±SD), while categorical variables were expressed as absolute and relative frequencies. Pearson’s correlation test assessed the correlation between BMD and TBS index. A chi-square test was used to compare the TBS classification distribution among the exams diagnosed as osteoporosis by REMS and DXA. The degree of concordance in classification by NOGG was assessed by calculating the percentage of participants classified in the same category (high risk or low risk) and applying Cohen’s kappa (k).

All statistical analyses were conducted using R software (4.2.0, R Core Team, 2022) and its packages ggplt2 and yardstick. *p* < 0.05 were considered statistically significant.

## RESULTS

**Figure 1:**
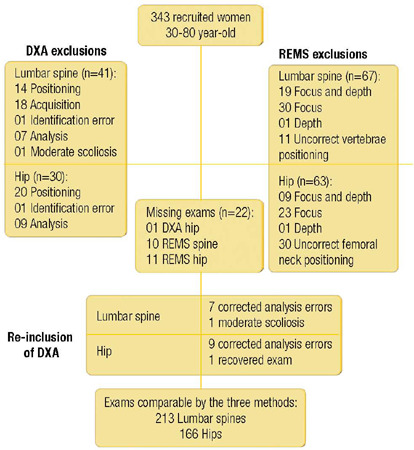
Flowchart demonstrating the selection of exams for the final analyses from the initial sample, with the reasons for exclusion for both methods.

A cohort of 343 female participants yielded 235 comparable lumbar spine exams by REMS, DXA, and 248 hip examinations, following the exclusion of exams due to insufficient quality. Twenty-two participants were excluded from the study due to BMI > 37 kg/m^2^ and age < 40 years. Additionally, 60 participants had only hip exams conducted using both methods. Consequently, 213 participants had comparable lumbar spine exams among the three methods, and 166 of them had comparable hip exams between REMS and DXA (Figure 1). The baseline characteristics of the participants are presented in Table 1.

**Table 1:** Baseline characteristics of the participants (mean ± SD or n (%))

Number of participants		n = 213
Age (years)		59.3 ± 8.6
BMI (kg/m^2^)		27.2 ± 4.3
Ethnicity	Asian	12 (5.7%)
	White	145 (68.0%)
	Black	33 (15.5%)
	Miscegenate	23 (10.8%)
Post-menopause		191 (89.7%)
With concomitant conditions (n = 71; 33.3%)	Rheumatoid arthritis	3 (1.4%)
	Diabetes mellitus 2	28 (13.1%)
	Current smoking	18 (8.5%)
	Use of glucocorticoid	15 (7.0%)
	Use of aromatase inhibitor	7 (3.3%)
Current use of antiresorptive agents		37 (17.4%)
Lumbar spine BMD (g/cm^2^) (n = 213)	REMS	0.843 ± 0.102
	DXA	0.876 ± 0.135
Femoral neck BMD (g/cm^2^) (n = 166)	REMS	0.697 ± 0.112
	DXA	0.729 ± 0.124
TBS		1.271 ± 0.111

**Figure 2:**
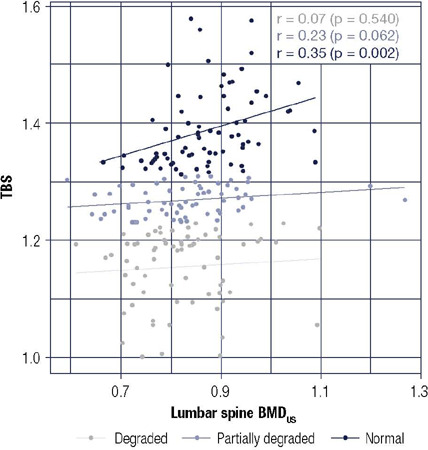
Correlation between lumbar spine BMD_US_ and TBS in the different TBS categories. TBS = trabecular bone score; BMD_US_ = bone mineral density by REMS.

**Figure 3:**
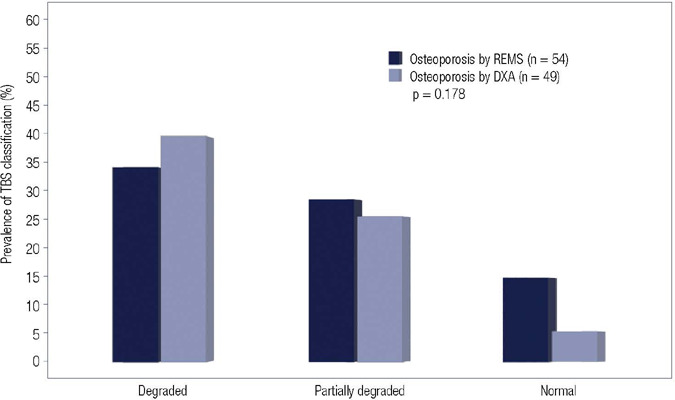
Distribution of TBS classifications among the lumbar spine exams classified as osteoporotic by REMS and DXA.

We observed a strong correlation (r = 0.74, *p* < 0.001) between BMD by REMS (BMD_US_) and BMD by DXA (BMD_DXA_) in the lumbar spine. However, both methods demonstrated a poor correlation with the TBS index (r = 0.27, *p* < 0.001 and r = 0.39, p < 0.001, for REMS and DXA, respectively). The poor correlation between BMD_US_ and TBS persisted even when the data were separated by category: normal, osteopenic, and osteoporotic. Only the values consistent with normality remained according to TBS (Figure 2). According to REMS analysis, the distribution of diagnosis on the lumbar spine exams was as follows: 54 (25.4%) osteoporosis, 123 (57.7%) osteopenia, and 36 (16.9%) normal. Of the 54 exams deemed osteoporotic at the lumbar spine by REMS, 24 (44.4%) were classified as degraded, 19 (35.2%) as partially degraded, and 11 (20.4%) as normal by TBS. Meanwhile, of the 49 exams considered osteoporotic at the lumbar spine by DXA, TBS classified 28 (57.1%) as degraded, 17 (34.7%) as partially degraded, and 4 (8.2%) as normal. The distribution of TBS classifications among REMS- and DXA-diagnosed osteoporotic exams was not statistically significantly different (*p* = 0.178) (Figure 3).

Most of the diagnostic discrepancies between REMS and DXA in lumbar spine exams occurred in scans classified as normal by DXA that were classified as osteopenia by REMS. Of the 72 normal DXA scans, 40 were classified as osteopenia by REMS (55.5%). The prevalence of TBS classification on these scans was normal in 20 cases (50%), partially degraded in 11 cases (27.5%), and degraded in 9 (22.5%).

Complete data for calculating absolute fracture risk by FRAX were obtained from 119 participants, of whom 13 (10.9%) reported a previous fragility fracture. The sites involved were vertebral (n = 4) and peripheral (n = 9). Those with reported fractures had significantly lower femoral neck BMD_DXA_ (p = 0.018), total hip BMD_DXA_ (*p* = 0.007), total hip BMD_US_ (*p* = 0.043), and TBS (*p* = 0.035) compared to those with no fractures. However, the distribution of the diagnoses obtained by each method did not significantly differ between groups.

Overall, a total of 92% of the participants received a concordant classification between REMS and DXA for major osteoporotic fracture risk with κ = 0.71 CI_95_% (0.54 to 0.89), and 87% were concordant for hip fracture risk with κ = 0.58 CI_95_% (0.38 to 0.77), with no significant differences between methods. Adjusting the fracture risk for TBS did not change the conclusions. Excluding those participants who were receiving an-tiresorptive medication also did not alter the results.

## DISCUSSION

According to these results, REMS performed similarly to DXA in classifying TBS bone degradation. Similarly, the intervention thresholds for osteoporosis treatment defined by the FRAX-NOGG strategy using the femoral neck T-score obtained by REMS were not different from those obtained using the DXA femoral neck T-score. Previously, we reported the high accuracy of REMS for diagnosing DXA-defined osteoporosis in this same sample of women (^16^).

The analyses seeking the association between REMS and TBS in the lumbar spine were performed because both are related to trabecular bone microarchitecture and should not be affected by lumbar osteoarthritis (^14^). However, the correlation between BMD_US_ and TBS in lumbar spine exams was low and consistent with low to moderate correlation between BMD_DXA_ and TBS already reported in other studies (^25,26,27^). Similarly, Fassio and cols. (^28^) found no correlation between REMS and TBS at the lumbar spine in a population with chronic kidney disease on peritoneal dialysis.

Also, considering that REMS assesses bone mass and quality (^8,10^), it was expected that there would be more TBS values between degraded and partially degraded in osteoporotic exams by REMS than in osteoporotic exams by DXA on the lumbar spines. However, there was no statistically significant difference between the two methods. Additionally, half of the exams classified as normal by DXA but osteopenic by REMS were classified as partially degraded or degraded by TBS, suggesting that REMS might be more assertive in identifying bone microarchitecture.

There was substantial agreement for major osteoporotic fracture risk and moderate agreement for hip fracture risk by applying the T-score values by REMS and DXA to the NOGG classification, with no statistically significant difference.

Adami and cols. (^9^) observed a slightly better performance for REMS than DXA regarding lumbar spine T-score, predicting participants presenting a fragility fracture at a follow-up. Interestingly, Cortet and cols. (^11^) found a slightly better performance for REMS than DXA for lumbar spine and femoral neck T-scores discriminating participants with previous osteoporotic fractures when studying more participants.

Recently, the REMS was applied to analyze clinical conditions wherein BMD_DXA_ is often misleading. Caffarelli and cols. (^12^) found that postmenopausal women with type 2 diabetes presented lower BMD_US _than BMD_DXA_ at all skeletal sites compared to the control group. They also found an inverse association between BMD_US_ at the lumbar spine and the disease duration. In addition, more participants with type 2 diabetes were diagnosed as osteoporotic by REMS than DXA. In another study, Caffarelli and cols. (^13^) evaluated young women with anorexia nervosa using REMS and DXA. The participants with previous vertebral fractures due to bone fragility compared to those without fractures showed a statistically significant lower total hip BMD_US_. Finally, Fassio and cols. (^28^) compared REMS and DXA bone analyses in participants undergoing peritoneal dialysis for chronic kidney disease, which resulted in more participants fulfilling the criteria for osteoporosis by DXA (43.6%) than REMS (32.4%) considering all sites. Therefore, REMS and DXA may not always have matching ratings, and more studies with populations and comorbidities are needed to establish these associations.

This study aimed to investigate the relationship between REMS analysis and trabecular microarchitecture integrity through comparisons with the TBS in a population of women in a real-life context: many ethnicities, a wide age range, primarily postmeno-pausal and presenting medical conditions related to bone loss. The particular importance of this study lies in showing similarities between REMS and DXA and identifying the discordant cases in which REMS may have detected the impaired bone quality shown by TBS. However, the present study has limitations: many exams of both methods were excluded. Additionally, 17.4% of the participants used antiresorptive agents, and TBS’s role in monitoring treatment is uncertain (^17^). Moreover, no published study has evaluated treatment monitored by REMS. Finally, as the fracture rate was low and only referred to, we did not use it for further analysis.

As a perspective, a software called Fragility Score has been recently developed for REMS to determine fracture risk independently from BMD_US_. It may be a similar tool to TBS for DXA. In addition, it may be helpful to identify which participants with osteoporosis by REMS should undergo a further radiological assessment to look for vertebral fractures.

Finally, REMS showed a high accuracy in diagnosing osteoporosis based on the gold standard DXA, as previously demonstrated in the sample of Brazilian women. The device is less expensive than a DXA densitometer, but there is still room for price reduction as it becomes more commercialized. In addition, it is portable, meaning it can be transported to different diagnostic centers covering larger geographic regions, increasing the population’s access to the exam and, consequently, to the diagnosis of osteoporosis. It is simpler to perform than DXA and does not require a physician for its analysis. As it does not contain ionizing radiation, there are no restrictions regarding radiological protection or technicians’ exposure. Therefore, based on this initial experience, REMS performs very close to DXA for the diagnosis of osteoporosis and the FRAX determination of intervention threshold. It may become an alternative for BMD measurements in regions inaccessible to DXA densitometry, increasing the population’s access to healthcare.

In conclusion, the BMD measured by REMS and DXA in the lumbar spines showed low correlations with the TBS index, and the distribution of each TBS classification among the osteoporotic lumbar spine exams by REMS and DXA was not statistically different. The FRAX risk probabilities calculated using REMS or DXA did not show statistical differences, which may be of interest for clinical practice. These findings suggest that REMS performs similarly to DXA and may become a surrogate method for diagnosing osteoporosis and fracture risk stratification using FRAX. Longitudinal studies will be essential to evaluate its ability to detect the effects of osteoporosis treatment on bone mass in the long term.
